# The Effects of Dietary *Arthrospira platensis* on Oxidative Stress Response and Pigmentation in Yellow Catfish *Pelteobagrus fulvidraco*

**DOI:** 10.3390/antiox11061100

**Published:** 2022-05-31

**Authors:** Cui Liu, Haokun Liu, Xiaoming Zhu, Dong Han, Junyan Jin, Yunxia Yang, Shouqi Xie

**Affiliations:** 1 State Key Laboratory of Freshwater Ecology and Biotechnology, Institute of Hydrobiology, Chinese Academy of Sciences, Wuhan 430072, China; liucui@ihb.ac.cn (C.L.); xmzhu@ihb.ac.cn (X.Z.); hand21cn@ihb.ac.cn (D.H.); jinjunyan@ihb.ac.cn (J.J.); yxyang@ihb.ac.cn (Y.Y.); sqxie@ihb.ac.cn (S.X.); 2 College of Advanced Agricultural Sciences, University of Chinese Academy of Sciences, Beijing 100049, China; 3 The Innovative Academy of Seed Design, Chinese Academy of Sciences, Beijing 100101, China; 4 Hubei Engineering Research Center for Aquatic Animal Nutrition and Feed, Wuhan 430072, China

**Keywords:** aquaculture, Spirulina, antioxidant, redox enzyme

## Abstract

In aquaculture, fish are often exposed to several stress conditions, which will cause oxidative disorder and bring about health and quality problems. *Arthrospira platensis* contains abundant bioactive ingredients, which are beneficial for animal health. This study was conducted to investigate the effects of *A. platensis* on pigmentation, antioxidant capacity, and stress response after air exposure of fish. A total of 120 yellow catfish *Pelteobagrus fulvidraco* (initial weight 70.19 ± 0.13 g) were divided into three tanks per treatment and fed diets supplemented with 0 g kg^−1^ *A. platensis* (CON) and 20 g kg ^−1^ *A. platensis* (AP) for 65 days. The results indicated that dietary *A. platensis* had no effects on the growth of yellow catfish. The AP diet significantly reduced lactic acid (LD) and cortisol levels stimulated by air exposure stress (*p* < 0.05). Dietary *A. platensis* significantly increased plasma superoxide dismutase (SOD) and glutathione peroxidase (GPX) activities and glutathione (GSH) contents, and the relative expression levels of *sod* and *cat,* to protect against oxidative stress caused by air exposure (*p* < 0.05). The AP diet significantly improved the relative expression level of *nrf2* (nuclear factor erythroid-2 related factor 2), while the relative expression level of *keap1* (kelch-like ECH associated protein 1) was downregulated, and the protein levels of liver Nrf2 were significantly increased after air exposure stimuli (*p* < 0.05). Dietary *A. platensis* significantly increased skin lutein contents, increased skin redness, yellowness and chroma (*p* < 0.05), and improved body color abnormalities after oxidative stress caused by air exposure stimuli. Skin yellowness was associated with lutein contents and the expression levels of some antioxidant genes to varying degrees. Overall, dietary *A. platensis* could be utilized as a feed additive to activate the antioxidant response, as well as alleviate oxidative stress and pigmentation disorder induced by air exposure.

## 1. Introduction

Stress is a general term that applies to a situation in which a person or an animal is subjected to a challenge that may result in real or symbolic danger to its integrity [1]. In aquaculture, fish are often exposed to several stress conditions due to farming practices (high densities, transport or handling, or changes in abiotic factors) and environmental factors (temperature, salinity, biochemical water quality, etc.) [2]. The response to stress in fish is often characterized as primary, which is the activation of the hypothalamic–pituitary–interrenal axis that eventually results in a hormonal response to the release of cortisol and catecholamine; secondary, which includes the changes in metabolic, hematological, hydromineral, and structural due to the action of cortisol and catecholamine; and tertiary, which are associated with the performance of the organism, exemplified by inhibited growth, hampered reproduction, and immunosuppression [3,4]. Extreme conditions also produce reactive oxygen species (ROS), which lead to DNA, protein, and lipid damage [5].

Air exposure is a common acute stressor with a subsequent physiological disturbance in aquaculture since handling procedures during animal husbandry and the production cycle require exposing fish to air environments [2]. Air exposure always brings a series of problems to aquatic animals, like exacerbating blood chemical conditions, causing oxidative and antioxidant responses, and adversely affecting survival [6,7,8,9]. In recent years, there has been increasing interest in using natural compounds as functional nutrients to reduce stress responses and improve the immunity of aquatic animals [10,11,12].

*Arthrospira platensis*, a blue-green alga that is gaining worldwide popularity as a food supplement, contains high contents of protein, polyunsaturated fatty acids, vitamins, and minerals [13]. In addition, *A. platensis* is plenitudinous in antioxidant compounds such as carotenoids, phycocyanin, and tocopherols [14]. In previous studies, it has been demonstrated that *A. platensis* can enhance antioxidant and immunity abilities as well as ensure skin pigmentation in fish [15,16,17].

Yellow catfish *Pelteobagrus fulvidraco* is a commercially important fish species in aquaculture in East and South Asia because of its excellent meat quality and perfect flavor [18,19]. However, farmed fish are always influenced by handling, weighing, crowding, grading, and transporting, which cause fish exposure to air and bring about a stress response. Hence, the main objectives of the study were to evaluate the antioxidant ability and stress responses of yellow catfish fed an *A. platensis*-supplemented diet and exposed to air as an experimental stressor causing oxidative stress. In addition, we also investigated the effect of *A. platensis* on the pigmentation of fish and detected the correlation between skin color and skin lutein content and oxidative stress. We are expecting that dietary *A. platensis* can reduce the stress responses and bring welfare to fish during fish farming.

## 2. Materials and Methods

### 2.1. Experimental Diets

Two isonitrogenous (420 g crude protein kg^−1^ diet) and isolipidic (80 g crude lipid kg^−1^ diet) diets were designed as practical diets, using white fishmeal, soybean meal, and rapeseed meal as blended protein sources (Table 1). This study consisted of a control group (CON-without supplementing *A. platensis*) and an *A. platensis* group (AP, 20 g kg^−1^ *A. platensis*). All ingredients were completely mixed with appropriate water and then made into pellets using an SLP-45 laboratory granulator (Fishery Mechanical Facility Research Institute, Shanghai, China). The diets were oven-dried at 60 °C for 12 h and stored at 4 °C until used for experimentation. Moisture, crude protein, crude lipid, and lutein contents of diets were analyzed following the same procedures as the previous study [20]. Moisture content was determined by oven drying at 105 °C to a constant weight. A 2300 Kjeltec Analyzer Unit machine (FOSS Tecator, Haganas, Sweden) was used to measure crude protein content. Crude lipid content was determined by ether extraction in a Soxtec System HT6 (Tecator Ltd., Haganas, Sweden). Lutein was extracted by mixed extractant (n-Hexane: acetone: ethanol: toluene = 50:35:30:35, *v*/*v*/*v*/*v*) and analyzed by HPLC.

### 2.2. Fish, Experimental Conditions, and Feeding Procedures

All experimental animal care protocols were approved by the ethics committee of the Institute of Hydrobiology, Chinese Academy of Sciences. Yellow catfish were obtained from the Dengjia State fish farm (Dengjiazhou, Jiangxia, Wuhan, China). Two weeks before the feeding trials, all fish were temporarily domesticated in a fiberglass cylinder (1500 L) and fed twice a day at 08:30 and 16:30. Feeding trials were conducted in an indoor recirculating system. At the beginning of each trial, the fish fasted for 24 h. Healthy and similarly sized fish (*n* = 120, 70.19 ± 0.13 g) were randomly selected, batch weighed, and placed into 6 fiberglass tanks (3 tanks per treatment, 20 individuals per tank, water volume, 400 L, diameter, 70 cm). The experimental fish were hand-fed to apparent satiation twice daily (8:30 and 16:30) for 65 days. Each tank was provided with continuous aeration. During the experiment, the water temperature was maintained at 30.5 ± 1.0 °C. Total ammonia-nitrogen was maintained at <0.1 mg L^−1^, dissolved oxygen at >6 mg L^−1^, and residual chloride at <0.01 mg L^−1^. The light period was from 8:00 to 20:00, and the light intensity was approximately 2.20–3.70 μmols^−1^m^−2^ (at water surface).

### 2.3. Sample Collection and Air Exposure Stress Challenge

At the end of the feeding trial, the experimental fish were batch-weighed. Three fish from each tank were randomly selected and anesthetized with 80 mg L^−1^ MS-222 (Sigma-Aldrich, St. Louis, MO, USA) and then measured coloration and sampled for blood, liver, kidney, dorsal, and abdominal skin tissues. Blood samples were collected from the caudal vein with heparinized syringes. After centrifugation (3500× *g*, 15 min, 4 °C), plasma was collected and stored at −80 °C for further analysis. After blood sampling, the liver, kidney, dorsal, and abdominal skin were dissected on ice and stored at −80 °C.

At the end of the trial, three fish were randomly selected from each tank for a stress challenge. Fish were exposed to air for 5 min and then sampled for blood, liver, and kidney. The methods of plasma collection and sample storage were the same as those for normal sampling (without air exposure).

### 2.4. Fish Skin Color Determination

The dorsal and abdominal skin color parameters of the fish were measured by a Konica Minolta CR-400 tristimulus colorimeter (Minolta, Osaka, Japan). The L* value represents lightness (0 for black, 100 for white), a* represents the red/green dimension, and b* represents the yellow/blue dimension, the value of chroma C is the distance from the lightness axis (L*) and starts at 0 in the center, in accordance with the recommendations of the International Commission on Illumination [21]. Chroma is an expression of the saturation or intensity of the color and is calculated by the equation C = (a*^2^ + b*^2^)^1/2^ [22]. Fish from different experimental groups were photographed using a Nikon D5100 camera (Japan).

### 2.5. Lutein Extraction and Quantification

The extraction of lutein from the skin was determined by following the method described previously [23]. Skin samples (200–300 mg) were separately mixed into a 0.7 mL solution consisting of 5% sodium chloride; 1 mL ethanol was added to homogenize the samples. During homogenization, 2 mL hexane was added. Then, the samples were centrifuged (4 °C, 4600 rpm, 10 min), and the hexane phase was collected. Extraction with hexane was performed twice, and the combined phase was evaporated under nitrogen to obtain pigment samples. The analytical conditions of lutein were based on those reported previously [24,25] with some modifications. The pigment samples were dissolved in an isocratic solvent system, methanol/methyl-tert-butyl ether = 86/14 (*v*/*v*), the lutein standard (07168, sigma) was diluted into 100, 50, 20, 10, 5, 2, 1, 0.5, 0.1 µg/mL with the same solvent system. The obtained solution was used for HPLC (Waters e2695, Milford, Delaware, USA) analyses immediately after passing through a 0.22 µm membrane filter. The HPLC was equipped with a Waters YMC Carotenoid C30 column (5 µm, 4.6 × 250 mm); the mobile phase consisted of solvent A (methanol: methyl-tert-butyl ether: H_2_O = 81:15:4) and solvent B (methanol: methyl-tert-butyl ether = 600:90). The gradient procedure was performed at a flow rate of 1 mL/min. The total run time was 40 min, and the injection volume was 10 µL.

### 2.6. Plasma LD, Cortisol, Glucose, and Antioxidant Assays

The contents of lactic acid (LD), cortisol, glucose (GLU), malondialdehyde (MDA), and reduced glutathione (GSH) and the activities of superoxide dismutase (SOD) and glutathione peroxidase (GPX) were tested using commercial kits (Nanjing Jiancheng Bioengineering Institute, Nanjing, Jiangsu, China).

### 2.7. Quantitative Real-Time PCR Analysis

Total RNA was extracted from the liver and kidney using TRIzol reagent according to the manufacturer’s instructions (Invitrogen, Carlsbad, CA, USA). The quality of total RNA was evaluated by 1% agarose gel electrophoresis. The purity and concentration were assessed by a NanoDrop^®^ ND-2000 UV–Vis Spectrophotometer (NanoDrop Technologies, Wilmington, DE, USA). The total RNA was then reverse-transcribed with an M-MLV FirstStrand Synthesis Kit (Invitrogen, Shanghai, China). The polymerase chain reaction (PCR) primer sequences for *sod*, *gpx*, *gr*, *cat*, *nrf2*, *keap1,* and the reference gene β-actin, which were designed based on the cDNA sequences of yellow catfish, are shown in Table 2. A LightCycler 480 System (Roche, Germany) with SYBR^®^ Green I Master Mix (Roche, Germany) was used to perform quantitative RT–PCR. Each sample was run in duplicate, and the relative expression was calculated [26].

### 2.8. Western Blot Analysis

Liver tissues were lysed with RIPA lysis buffer (Beyotime Biotechnology, China) containing protease inhibitor cocktail and phosphatase inhibitor cocktail (Roche, Basel, Switzerland). Equal amounts of protein were separated on sodium dodecyl sulfate–polyacrylamide gel electrophoresis (SDS–PAGE) gels and transferred to polyvinylidene fluoride (PVDF) membranes. The PVDF membranes were blocked for 1 h with 5% milk in TBST buffer and then incubated overnight at 4 °C with Nrf2 (1:1000, ab62352, Abcam) and β-actin (1:1000, #8457, CST) antibodies. β-actin was used as an internal reference protein. Horseradish peroxidase-labelled secondary antibodies were used to generate a chemiluminescent signal that was detected by ImageQuant LAS 4000 mini (GE Healthcare Life Sciences) and quantified using ImageJ software (National Institutes of Health).

### 2.9. Statistical Analyses

All data were statistically analyzed with SPSS 19.0 and subjected to one-way ANOVA. Before any statistical analysis, normality and homoscedasticity assumptions were confirmed. Duncan’s multiple range test was used to detect the significance of differences in mean values among different treatments. The results are presented as the mean ± standard error (SEM). The Pearson’s correlation coefficient was calculated to analyze the significance of linear relationships between skin yellowness (b*) and the studied variables. The significance difference level was set at *p* < 0.05.

## 3. Results

### 3.1. Growth and Feed Utilization

The survival rate of fish was 100% in both groups during the feeding trial; there were no significant differences in final body weight (FBW), specific growth rate (SGR), feeding rate (FR), or feed efficiency (FE) in either the CON or AP groups (Table 3). At the end of the experimental period, no significant differences were found in the condition factor, hepatosomatic index, or viscerosomatic index between the CON and AP groups (Table 3).

### 3.2. Stress Response Markers in Plasma

**Figure 1:**
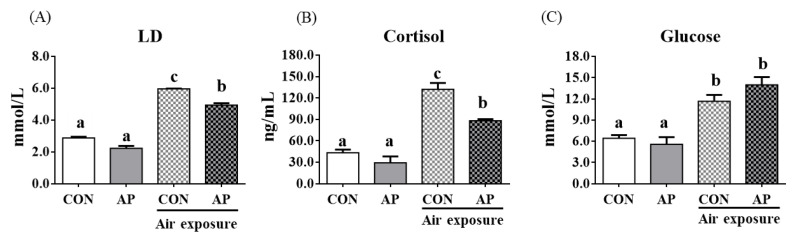
Plasma stress biomarkers in the two groups before and after air exposure stress. LD (**A**), cortisol (**B**), and glucose (**C**). Values are represented as the mean ± SEM (*n* = 6). Bars with different lowercase letters mean significant differences among groups (*p* < 0.05).

Stress response markers such as LD, cortisol, and glucose were significantly increased after air exposure stress in fish fed the CON diet (*p* < 0.05, Figure 1). However, the AP diet significantly reduced LD and cortisol levels stimulated by air exposure stress (*p* < 0.05, Figure 1).

### 3.3. Antioxidant-Related Parameters in Plasma

**Figure 2:**
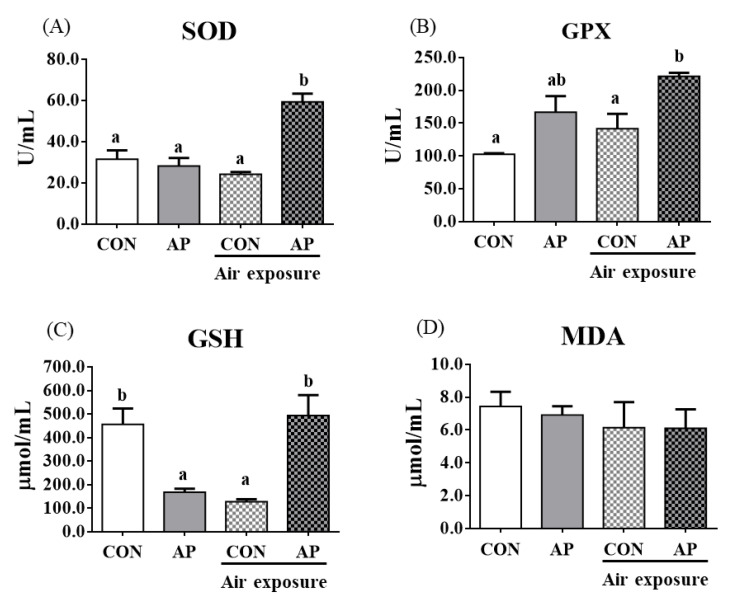
Plasma antioxidant related parameters in the two groups before and after air exposure stress. SOD (**A**), GPX (**B**), GSH (**C**), and MDA (**D**). Values are represented as the mean ± SEM (*n* = 6). Bars with different lowercase letters mean significant differences among groups (*p* < 0.05).

To test whether dietary AP affected fish antioxidant capacity, we analyzed antioxidant-related parameters in plasma. As shown in Figure 2, the AP diet significantly increased plasma SOD and GPX activities after air exposure to protect against oxidative stress (*p* < 0.05). The contents of plasma GSH were significantly decreased in the CON group after air exposure stimuli (*p* < 0.05), while the AP diet significantly improved GSH contents to protect against oxidative stress caused by air exposure (*p* < 0.05). The MDA content showed no obvious changes.

### 3.4. Antioxidant Related Gene Expression and the Nrf2 Signaling Pathway in the Liver

**Figure 3:**
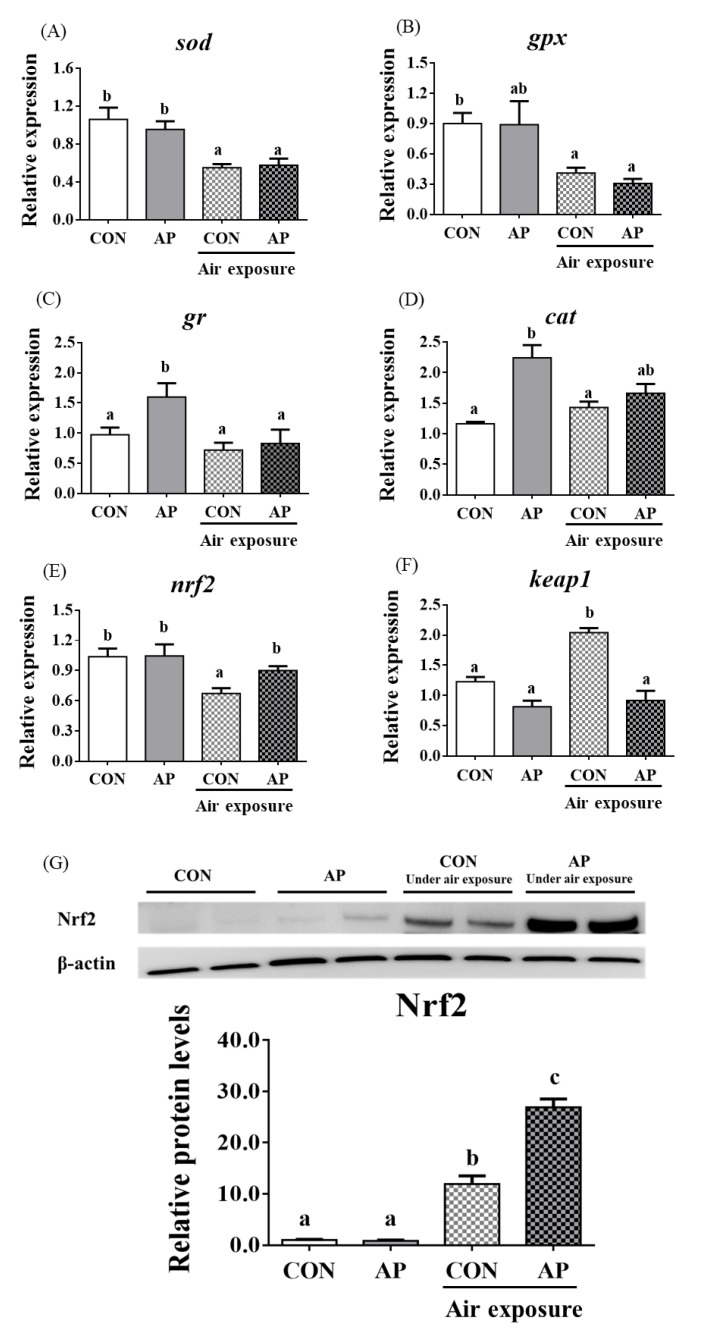
*sod* (**A**), *gpx* (**B**), *gr* (**C**), *cat* (**D**), *nrf2* (**E**), and *keap1* (**F**) gene relative expressions levels and Nrf2 (**G**) protein relative expressions level of liver in the two groups before and after air exposure stress. Data are indicated as mean ± SEM (*n* = 6). Bars with different lowercase letters mean significant differences among groups (*p* < 0.05).

As presented in Figure 3, the relative expression levels of liver *sod* and *gpx* were significantly downregulated in the CON group after air exposure stimulation (*p* < 0.05), and there were no obvious differences in the relative expression levels of liver *gr* and *cat* between CON and AP groups after air exposure stimulation.

The relative expression level of *nrf2* in the liver was significantly downregulated in the CON group after air exposure stimulation (*p* < 0.01), while the AP diet significantly improved the relative expression level of *nrf2* to protect against oxidative stress caused by air exposure stimuli (*p* < 0.05, Figure 3). The relative expression level of *keap1* in the liver was significantly upregulated in the CON group after air exposure stimulation (*p* < 0.05), while the AP diet significantly reduced the relative expression level of *keap1* to protect against oxidative stress caused by air exposure (*p* < 0.05, Figure 3). The protein levels of liver Nrf2 were significantly higher in the AP group than that in the CON group after air exposure stimulation (*p* < 0.01, Figure 3).

### 3.5. Antioxidant Related Gene Expression in Kidney

**Figure 4:**
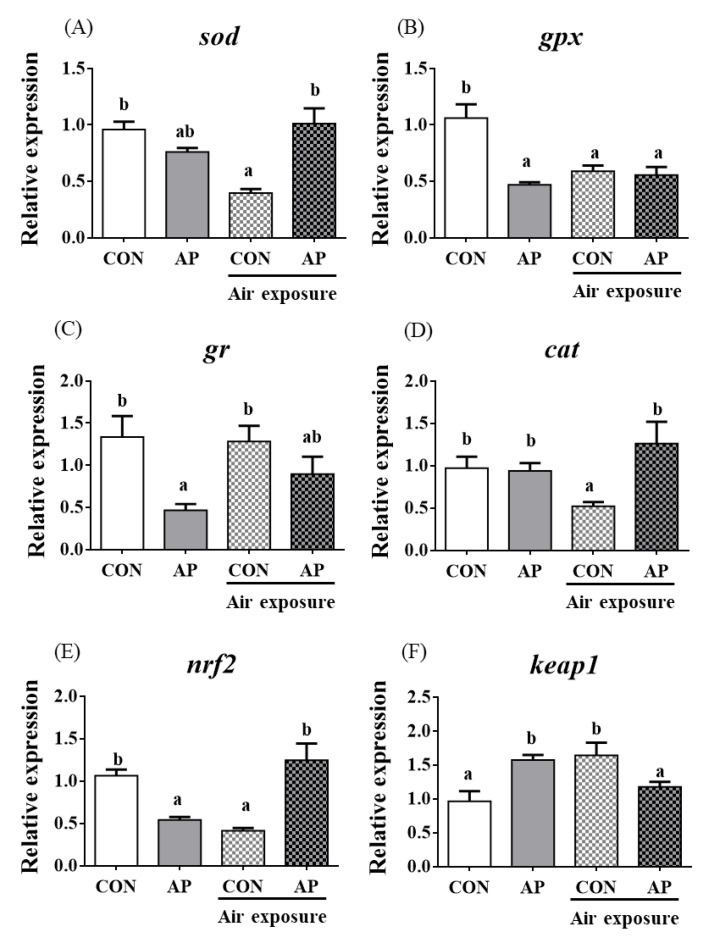
*sod* (**A**), *gpx* (**B**), *gr* (**C**), *cat* (**D**), *nrf2* (**E**), and *keap1* (**F**) gene relative expressions levels of kidney in the two groups before and after air exposure stress. Data are indicated as mean ± SEM (*n* = 6). Bars with different lowercase letters mean significant differences among groups (*p* < 0.05).

The relative expression levels of *sod*, *gpx,* and *cat* were significantly downregulated in the CON group after air exposure stimulation (*p* < 0.05, Figure 4); however, the AP diet significantly improved the relative expression levels of *sod* and *cat* to protect against oxidative stress caused by air exposure (*p* < 0.05) but did not significantly affect the relative expression levels of *gr* in both groups after air exposure stimulation (Figure 4).

The relative expression level of *nrf2* in the kidney was significantly downregulated in the CON group after air exposure stimuli (*p* < 0.01), while the AP diet significantly improved the relative expression level of *nrf2* to protect against oxidative stress caused by air exposure stimuli (*p* < 0.05, Figure 4). The relative expression level of *keap1* in the kidney was significantly upregulated in the CON group after air exposure stimuli (*p* < 0.05), while the AP diet significantly reduced the relative expression level of *keap1* to protect against oxidative stress caused by air exposure (*p* < 0.05, Figure 4).

### 3.6. Skin Lutein Content and Body Color of Fish

**Figure 5:**
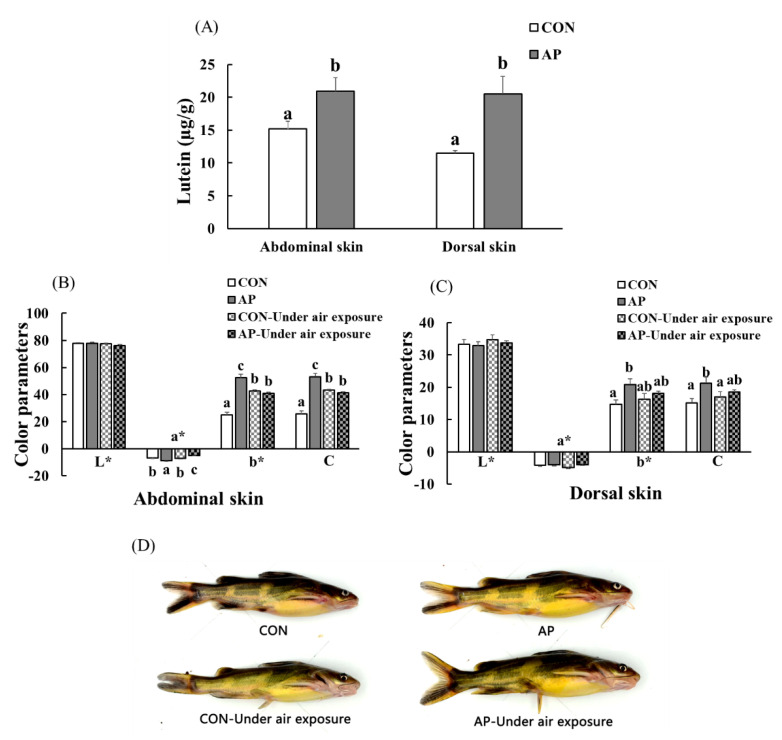
The lutein contents in the skin of yellow catfish fed on different experimental diets (**A**), color parameters of abdominal (**B**) and dorsal skin (**C**), and body color of fish (**D**) in the two groups before and after air exposure stress. Data are indicated as mean ± SEM (*n* = 6). Bars with different lowercase letters mean significant differences among groups (*p* < 0.05).

The lutein contents of the Con and SP diets were 4.96 and 8.08 µg g^−1^, respectively. At the end of the feeding trial, the abdominal and dorsal skin lutein contents increased significantly in fish fed the AP diet (*p* < 0.05, Figure 5A).

As shown in Figure 5, there was no significant difference in skin lightness (L*) among all groups. At the end of the experimental period, dietary *A. platensis* supplementation significantly increased the abdominal skin redness value (*p* < 0.05), and air exposure led to a significantly decreased abdominal skin redness value (*p* < 0.05). Skin yellowness and chroma were significantly higher in catfish fed the AP diet at the end of the feeding trial (*p* < 0.05). In the CON group, the abdominal skin yellowness and chroma values of fish were significantly increased after air exposure, while the abdominal skin yellowness and chroma values of fish were significantly decreased in the AP group after air exposure (*p* < 0.05), and fish fed the AP diet had clearer black spots and saturated yellow coloration both before and after air exposure.

### 3.7. Skin Yellowness Correlation Analysis

A positive and strong correlation between skin yellowness and skin lutein contents was detected (*p* < 0.05, Table 4). We found a negative but not very strong correlation between skin yellowness and plasma LD content (*p* < 0.05, Table 4). Abdominal skin yellowness presented a positive but not significant correlation with liver *sod* and *gr* relative mRNA levels, while dorsal skin yellowness presented a very positive and significant correlation with liver *sod* and *gr* relative mRNA levels (*p* < 0.05, Table 4). Positive and significant correlations were observed between abdominal skin yellowness and kidney *gpx* relative mRNA levels (*p* < 0.05), while positive but nonsignificant correlations were observed between dorsal skin yellowness and kidney *gpx* relative mRNA levels (Table 4). Abdominal skin yellowness presented a positive but not significant correlation with kidney *gr* relative mRNA levels, while dorsal skin yellowness presented a strong positive and significant correlation with kidney *gr* relative mRNA levels (*p* < 0.05, Table 4).

## 4. Discussion

The present study supported that air exposure induced oxidative stress in fish. Oxidative stress usually causes health and quality problems in fish. Our previous research showed that *A. platensis* can enhance the antioxidant and immune response capacities of fish [17,20]. In the present study, we focused on investigating whether dietary *A. platensis* could alleviate oxidative stress and disordered pigmentation caused by air exposure stimulation in fish.

### 4.1. A. platensis Did Not Affect the Growth Performance and Feed Utilization of Fish

The yellow catfish is an omnivorous freshwater fish [20], which, when fed on diets supplemented with *A. platensis,* did not exhibit differences in growth and feed efficiency. These results are in agreement with a previous study [17]. The previous study also indicated that the dietary inclusion of 25 g kg^−1^ *A. platensis* did not influence the growth of the great sturgeon *Huso huso*; however, higher content of diet *A. platensis* could effectively improve growth performance [27]. We demonstrated that *A. platensis* in the diets of yellow catfish resulted in an apparent digestibility coefficient (ADC) of dry matter and protein up to 70% and 90%, respectively [20]. The above results further validated that it is acceptable to supplement *A. platensis* in yellow catfish diets.

### 4.2. A. platensis Enhanced Stress Response

Cortisol is regarded as a primary physiological stress response in fish [28], and glucose and lactic acid will increase during stress responses [29]. In this study, the plasma cortisol levels increased significantly in yellow catfish due to exposure to the air for 5 min, with similar increases in plasma glucose and lactic acid levels. Air exposure is a stress factor for fish, which is in agreement with the previous studies [30,31]. Notably, the present study showed that *A. platensis* supplementation was effective against air exposure-induced stress responses by reducing plasma cortisol and lactic acid levels. In addition, the reduction of stress markers was dose-dependent on the *A. platensis* supplement [14].

### 4.3. A. platensis Enhanced the Antioxidant Capacity of Fish

The antioxidant enzymes SOD and GPX and the nonenzymatic antioxidant GSH play important roles in free radical scavenging to protect against oxidative damages [32]. In our study, dietary *A. platensis* increased the activities of SOD and GPX and GSH contents to protect against oxidative stress caused by air exposure stimuli. *A. platensis* enhanced antioxidant enzyme activities to protect fish from stress [15,33]. β-carotene, phycocyanin, algal polysaccharides, and polyphenol from *A. platensis* might work to activate antioxidant response [34,35,36,37].

Nrf2 plays a critical role in antioxidative defense responses [38]. Usually, *nrf2* transcription is inhibited by binding to *keap1*; when the *keap1*/*nrf2* complex is activated, *nrf2* is translocated into antioxidative factors in the nucleus, where it can regulate antioxidant enzymes, such as *sod*, *cat,* and *gpx* [39,40,41]. In the present study, the gene and protein expression levels of *nrf2* after air exposure were efficiently upregulated by *A. platensis* supplementation. Concurrently, the expression levels of *sod* and *cat* were obviously activated to improve the antioxidant status of yellow catfish, which is in accordance with a previous study [17]. *A. platensis* involves various proteins, which could have biological activities to induce *nrf2* stabilization and antioxidative enzymes [42]. Additionally, another component of *A. platensis*, polyphenol, has been found to modulate Nrf2-mediated antioxidant events [43,44]. Hence, *A. platensis* can activate the Nrf2 signaling pathway and may be advantageous for its use as an antioxidant nutritional supplement in aquatic animals.

### 4.4. A. platensis Improved Body Color Disorder Caused by Oxidative Stress

*A. platensis* supplementation enhanced the body color of fish at the end of the experimental trial in accord with previous studies [45,46]. It is known that fish need carotenoids to maintain normal body color from feed [47]. *A. platensis* is considered a carotenoid-producing organism, and lutein and zeaxanthin are mainly found in *A. platensis* [48,49]. The carotenoid pigments of *A. platensis* can also enhance the natural mucus layer, which is responsible for maintaining the glowing appearance of skin in fish [45]. The present results are in accordance with previous studies, suggesting that *A. platensis* is a proper color enhancer.

In this study, *A. platensis* addition promoted increased lutein contents in the skin, and we demonstrated that lutein is mainly deposited in the skin of yellow catfish earlier [50]. This study also confirmed the results. The correlational approach used in this study showed that skin yellowness (b*) was significantly associated with skin lutein contents. Lutein belongs to the xanthophylls, which are a type of carotenoid with antitumor and anti-inflammatory activities. Due to their chemical structure being rich in double bonds that provide them with antioxidant properties, lutein can protect other molecules from oxidative stress by turning off singlet oxygen damage through various mechanisms [51].

Skin color change has the potential to be a useful real-time indicator of stress [52]. Our research also found that skin yellowness was associated with plasma lactate. Notably, stress-induced responses of abnormal body color fish were stronger than those of normal fish, and the albino European catfish *Silurus glanis* showed more obvious behavioral and physiological responses to short-term stress induced by a combination of air exposure and novel environmental stressors than pigmented fish [53]. In this study, dietary *A. platensis* improved body color abnormalities after oxidative stress caused by air exposure stimuli, and skin yellowness was associated with the expression levels of some antioxidant genes to varying degrees. The results may suggest that the antioxidant components (such as lutein) in *A. platensis* successfully help fish defend against body color disorders caused by oxidative stress.

## 5. Conclusions

Our overall results demonstrated that 20 g kg^−1^ dietary *A. platensis* had no negative effects on the growth of fish but could reduce LD and cortisol levels, increase the activities of antioxidant enzymes, upregulate the expression levels of certain antioxidant-related genes, and regulate the Nrf2 signaling pathway, which aimed to protect against oxidative stress caused by air exposure stimuli. In addition, *A. platensis* could alleviate body color disorder caused by air exposure stress by increasing skin lutein contents. In terms of cost-benefit, the inclusion of 20 g kg^−1^ dietary *A. platensis* can enhance the ability of antioxidants and improve the body color of yellow catfish, as a result, the risk of suffering disease and farming losses will be reduced and market value will be enhanced. Moreover, in our previous study, the *A. platensis* can replace fishmeal in the diets of yellow catfish [20]. With global fisheries approaching unsustainable limits, current fishmeal production will inadequately support the cost-effective demands of aquafeeds; *A. platensis* could be a potential protein source to replace fishmeal in the future.

## Figures and Tables

**Table 1:** Diet formulation and chemical compositions of the experimental diets.

Ingredient (g kg^−1^ Dry Matter)	CON	AP
White fishmeal ^1^	300	300
*A. platensis * ^2^	0	20
Soybean meal ^3^	187.8	175.8
Rapeseed meal ^3^	200	185.5
Wheat flour	150	150
Fish oil	25	25
Soybean oil	25	25
Cellulose	27.2	33.7
Vitamin premix ^4^	3.9	3.9
Mineral premix ^5^	50	50
CMC ^6^	30	30
Choline chloride	1.1	1.1
Chemical composition (g kg^−1^)		
Moisture	108.55	108.15
In dry matter (g kg^−1^)		
Crude protein	418.30	416.56
Crude lipid	80.99	75.22
Lutein (µg/g)	4.96	8.08

^1^ White fishmeal: Seafood white fishmeal imported from the United States. ^2^* A. platensis*: Center for Microalgal Biotechnology and Biofuels, Wuhan, China. ^3^ Soybean meal and rapeseed meal were purchased from Wuhan Gaolong Feed Co., Ltd., Wuhan, Hubei, China. ^4^ Vitamin premix (mg kg^−1^ diet): Vitamin B_1_, 20; Vitamin B_2_, 20; Vitamin B_6_, 20; Vitamin B_12_, 0.02; folic acid, 5; calcium pantothenate, 50; inositol, 100; niacin, 100; biotin, 0.1; cellulose, 3522; Vitamin A, 11; Vitamin D, 2; Vitamin E, 100; Vitamin K, 10. ^5^ Mineral premixes (mg kg^−1^ diet): NaCl, 500.0; MgSO_4_·7H_2_O, 8155.6; NaH_2_PO_4_·2H_2_O, 12,500.0; KH_2_PO_4_, 16,000.0; Ca(H_2_PO_4_) 2H_2_O, 7650.6; FeSO_4_·7H_2_O, 2286.2; C_6_H_10_CaO_6_·5H_2_O, 1750.0; ZnSO_4_·7H_2_O, 178.0; MnSO_4_·H_2_O, 61.4; CuSO_4_·5H_2_O, 15.5; CoSO_4_·7H_2_O, 0.91; KI, 1.5; Na_2_SeO_3_, 0.60; Corn starch, 899.7. ^6^ CMC: Carboxymethyl cellulose.

**Table 2:** Sequences of the primers used for qRT-PCR analysis in yellow catfish.

Gene	Forward Primer (5′–3′)	Reverse Primer (5′–3′)	Amplicon Size (bp)	Tm (°C)	PCR Efficiency ^a/b^	Accession No
*sod*	TTGGAGACAATACAAATGGGTG	CATCGGAATCGGCAGTCA	129	57	2.007/1.968	XM_027171881
*gpx*	ATCTACATTGGCTTGGAAAC	GAAAGTAGGGACTGAGGTGA	257	58	1.966/1.950	XM_027163146
*gr*	CAGTCGCTTTGTTTGTTCTA	TCCTCCGATACACTTCTCAC	280	57	1.992/2.050	XM_027152663
*cat*	TCTGTTCCCGTCCTTCATCC	ATATCCGTCAGGCAATCCAC	151	58	1.964/2.001	XM_027163801
*nrf2*	TCTCGCCCAGTTACAGCTTG	GTTCCGTGAACGCCACATTC	128	60	1.992/1.952	XM_027164284
*keap1*	CGCAGCCGGGCTTTTATTTT	AGGCAGAAACGGGTTCAAGT	286	59	1.989/1.959	XM_027133478.1
*β-actin*	TTCGCTGGAGATGATGCT	CGTGCTCAATGGGGTACT	136	58	2.044/2.003	EU161066

^a^ Liver, ^b^ Kidney. *sod*, superoxide dismutase; *gpx*, glutathione peroxidase; *gr*, glutathione reductase; *cat*, catalase; *nrf2*, nuclear factor erythroid-2 related factor 2; *keap1*, kelch-like ECH associated protein 1.

**Table 3:** Growth, feed utilization, and morphological indices of yellow catfish fed different experimental diets.

Indices/Diets	CON	AP
IBW (g)	69.8 ± 0.10	70.23 ± 0.15
FBW (g)	103.23 ± 0.35	105.32 ± 1.07
Survival rate (%)	100	100
FR (%BW d^−1^)	2.52 ± 0.08	2.63 ± 0.02
SGR (% d^−1^)	0.68 ± 0.05	0.62 ± 0.02
FE (%)	30.51 ± 3.82	30.69 ± 0.49
Condition indices		
Condition factor (g cm^−3^)	1.89 ± 0.08	1.81 ± 0.04
Hepatosomatic index (%)	1.40 ± 0.17	1.68 ± 0.11
Viscerosomatic index (%)	11.14 ± 0.85	10.8 ± 0.56

IBW: initial body weight. FBW: final body weight. FR: feeding rate (% body weight day ^−1^) = 100 × (feed intake in dry matter)/[days × (initial body weight + final body weight)/2]. SGR: specific growth rate (% d ^−1^) = 100 × [ln (final body weight) − ln (initial body weight)]/days. FE: feed efficiency (%) = (final body weight-initial body weight)/feed intake in dry matter. Condition factor (g cm^−3^) = whole body weight/(body length)^3^ × 100. Hepatosomatic index (%) = liver weight/whole body weight × 100. Viscerosomatic index (%) = visceral weight/whole body weight × 100. Data are presented as the mean ± SEM (*n* = 3). No significant differences were found between two feeding experiments (*p* > 0.05).

**Table 4:** Correlation coefficients (r) between yellow catfish skin yellowness (b*-values) and skin lutein and oxidative stress response.

	Yellowness (b*)-Values	
	Abdominal Skin	Dorsal Skin
Dorsal skin lutein content	0.82 *	0.87 *
Abdominal skin lutein content	0.90 **	0.67 *
Plasma LD content	−0.59 *	−0.66 *
Liver *sod* relative mRNA level	0.76	0.95 *
Liver *gr* relative mRNA level	0.91	0.99 **
Kidney *gpx* relative mRNA level	0.96 *	0.99
Kidney *gr* relative mRNA level	0.80	0.98 *

* *p* < 0.05; ** *p* < 0.01.

## Data Availability

Data is contained within the article.
